# Supplementary material for: Dietary Emulsifiers Exacerbate Food Allergy and Colonic Type 2 Immune Response through Microbiota Modulation
Source: Nutrients. 2022 Nov 23;14(23):4983. doi: 10.3390/nu14234983 (PMC9738911; doi:10.3390/nu14234983)
Supplement: Supplementary file 1 [file nutrients-14-04983-s001.zip › nutrients-2031892-supplementary.pdf]

Supplementary Figure Legends

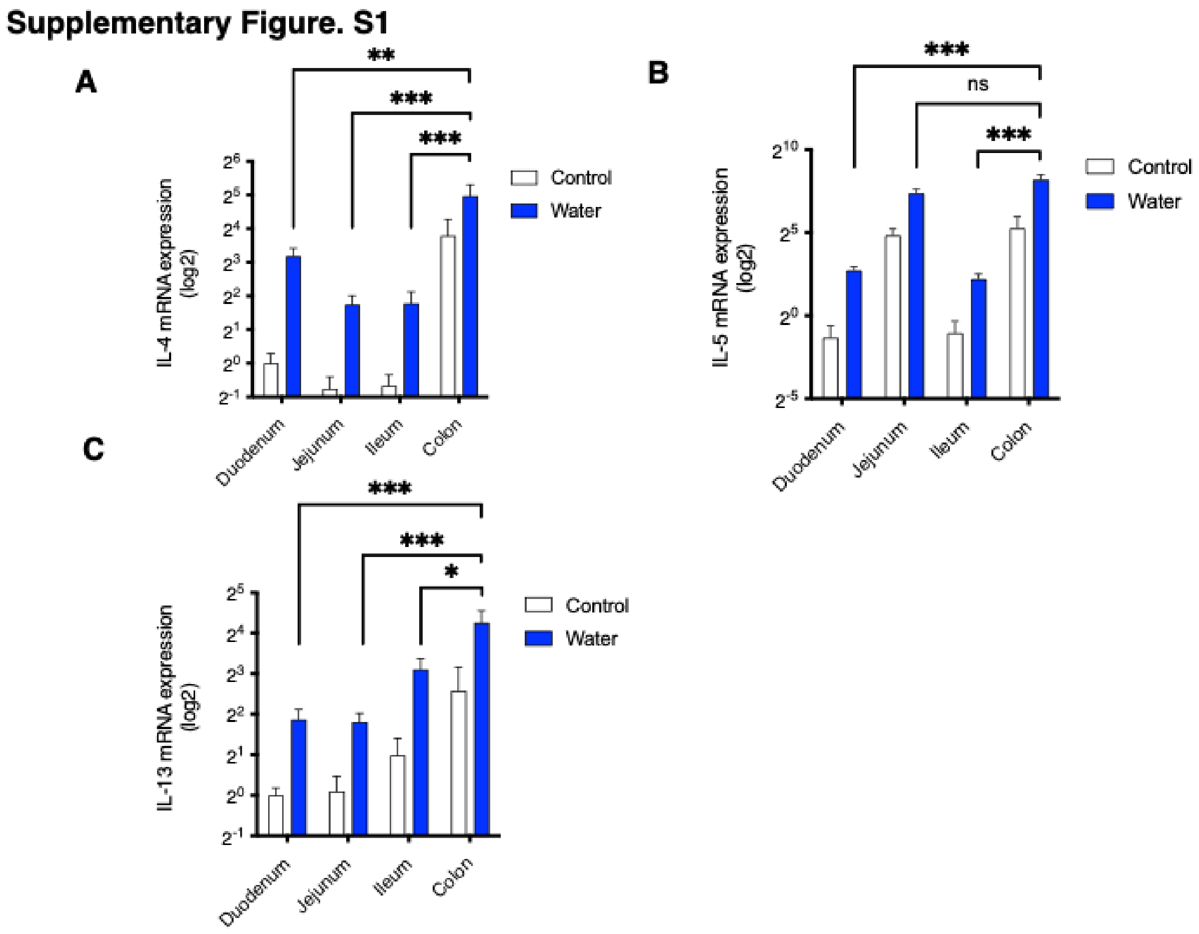

Figure S1. (**A–C**) The mRNA expression for IL-4, IL-5, and IL-13 in duodenum, jejunum, ileum, and colon by using quantitative PCR. The white bar represents the control group (sham) without food allergy induction; the blue bar represents the food allergy-induced group treated with water (no dietary emulsifier treatment). (Control, n = 5, Water; n = 7). Student's *t* test was used to determine significance. Data are the means ± SEM. * $P < 0.05$, ** $P < 0.01$, *** $P < 0.001$; ns, not significant.

Supplementary Figure. S2

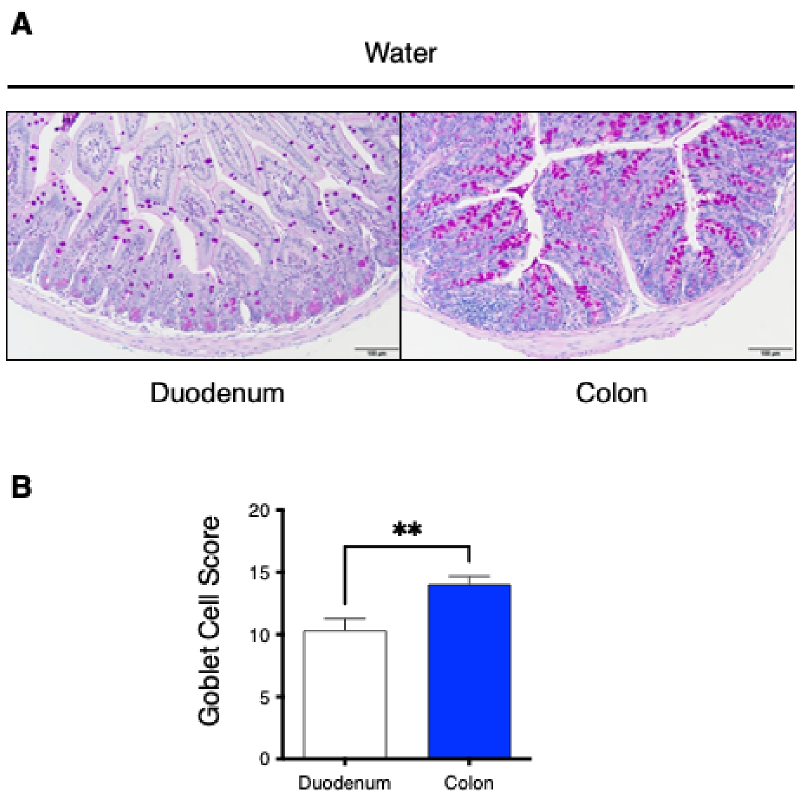

Figure S2. (**A**) Periodic acid-Schiff (PAS) staining for duodenal and colonic tissue in food allergy-induced mice (Water). (**B**) Goblet Cell Score of PAS-stained duodenal and colonic tissue as of A (n = 9 for duodenum; n = 18 for colon). Student's *t* test was used to determine significance. Data are the means ± SEM. ** $P < 0.01$; ns, not significant.

Supplementary Figure. S3

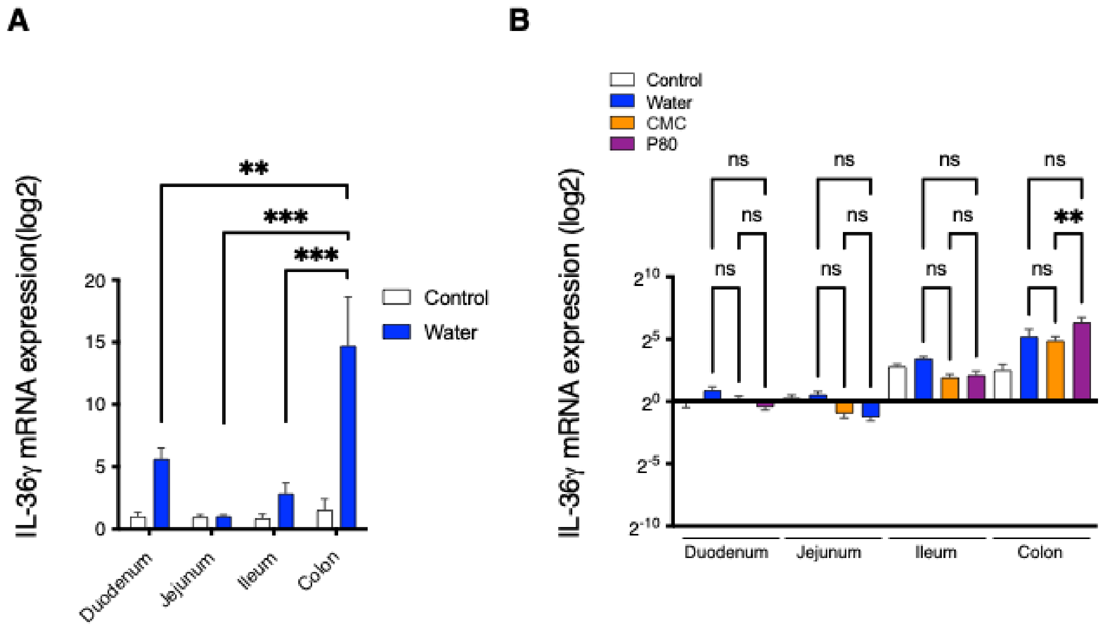

Figure S3. (**A**) The mRNA expression for IL-36γ in duodenum, jejunum, ileum, and colon by using quantitative PCR. The white bar represents the control group (sham) without food allergy induction; the blue bar represents the food allergy-induced group treated with water (Control, n = 5, Water; n = 7) (**B**) The mRNA expression for IL-36γ in duodenum, jejunum, ileum, and colon by using quantitative PCR. The white bar represents the control group without food allergy induction; the blue bar represents the food allergy-induced group treated with water (no dietary emulsifier treatment); the orange bar represents CMC treated group; the purple bar represents P80 treated group. (n = 5, Control; n = 10, Water; n = 9, CMC; n = 9, P80). Student's *t* test (**A**) or One-way ANOVA and Tukey's Multiple Comparison Test (**B**) were used to determine significance. Data are the means ± SEM. ** $P < 0.01$, *** $P < 0.001$; ns, not significant.
